# Tianeptine’s Obscured Withdrawal, Presentation, and Treatment

**DOI:** 10.7759/cureus.62554

**Published:** 2024-06-17

**Authors:** Amin Farsani, Carlo Reyes

**Affiliations:** 1 Emergency Medicine, Los Robles Regional Medical Center, Thousand Oaks, USA

**Keywords:** withdrawal, abuse, misuse, tricyclic antidepressant toxicity, tianeptine

## Abstract

The misuse of over-the-counter supplements containing the drug tianeptine poses significant public health concerns, as evidenced by a rise in Poison Control calls and social media discussions regarding its adverse effects. This case report highlights a 67-year-old Caucasian male presenting with symptoms suggestive of tianeptine withdrawal after consuming excessive doses of the supplements daily and their abrupt cessation 12 hours prior to arrival. Despite stable vitals, the patient exhibited anticholinergic toxidrome manifestations, necessitating supportive care and monitoring for acute intoxication. The patient's symptoms resolved, and he was discharged after two days in the hospital. Differential diagnosis complexities underscore the need for enhanced screening protocols and tailored treatment strategies. The discussion emphasizes the importance of prompt identification and management of tianeptine-related complications and calls for heightened awareness among healthcare providers.

## Introduction

Over-the-counter supplements containing the drug tianeptine have been misused by individuals in the United States to experience its euphoric effects by taking more than the recommended dose [1]. According to El Zahran et al., from 2014 through 2017, there was a statistically significant increase in calls to Poison Control related to the exposure and intentional abuse or misuse of these supplements, with the total number of tianeptine exposure calls rising from five in 2014 to 81 in 2017. From 2014 to 2020, there was a decline in social media discussions about the beneficial outcomes of tianeptine, accompanied by a rise in conversations about its negative consequences and withdrawal symptoms [2]. During this period, people turned to tianeptine for various reasons, including using it as a replacement or to alleviate withdrawal symptoms from other substances, self-medicating for psychiatric issues, and seeking enhancement in their quality of life, mood, or performance. Coingestants used for potentiated effects that are common with tianeptine are phenibut, ethanol, benzodiazepines, and opioids [1]. Government bodies and healthcare organizations are expressing worry over the inappropriate use of tianeptine, prompting deliberations regarding the potential implementation of scheduling or constraints on its accessibility [3]. 

Tianeptine is an atypical tricyclic antidepressant used in Europe, Asia, and Latin America to treat major depressive disorder (MDD) [1]. The resemblance to another tricyclic compound, amineptine, raises concerns about the potential amphetamine-like characteristics of tianeptine, and recent studies have pinpointed tianeptine as an effective stimulator of mu and delta opiate receptors [3]. The misuse of tianeptine presents a dilemma for healthcare professionals because its withdrawal symptoms have the potential to imitate those associated with other substances, such as opioids or anticholinergic medications. This has the potential to result in an incorrect diagnosis and unsuitable treatment.

## Case presentation

A 67-year-old Caucasian male with a significant medical history of polysubstance misuse, chronic hyponatremia, MDD, and anxiety was brought in by ambulance to the emergency department (ED) for lightheadedness and lethargy after stopping over-the-counter supplements containing tianeptine. The patient stated that he started taking the supplements to “get high” four weeks prior to his ED visit, consuming four to eight capsules daily that he purchased from a local smoke shop. His last dose was approximately 12 hours prior to his arrival. The patient tried to stop taking the supplements, but the withdrawal symptoms were unbearable. The patient endorsed constipation, urinary retention, chest pain in the morning, shortness of breath, palpitations, headaches, blurry vision, fevers, chills, and anorexia. 

**Figure 1:**
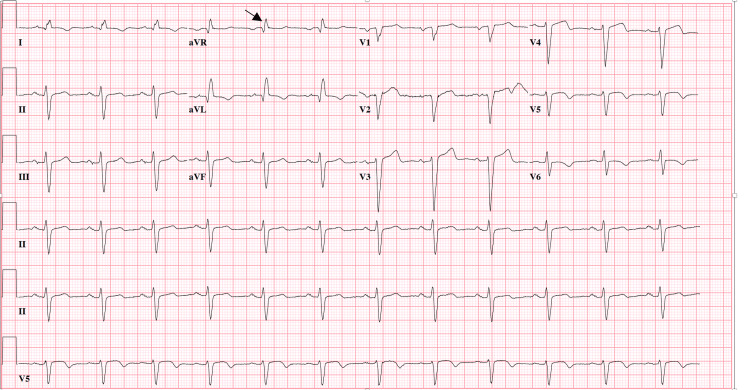
Initial ECG interpretation was as follows: normal sinus rhythm (NSR); left axis deviation (LAD); normal intervals except QRS prolonged at 120 m/s; R-wave > 3 mm in aVR (black arrow); T-wave inversions in lateral leads similar to prior ECG six years ago; Q waves in septal leads suggestive of old myocardial infarction (MI); no acute ST-segment elevation myocardial infarction (STEMI) or ischemia.

The patient's medication list was as follows: clopidogrel, carvedilol, ezetimibe, pravastatin, sacubitril/valsartan, aspirin, vilazodone, and Xanax. On examination, the patient’s vitals were stable, mydriasis was present, he was alert and oriented to person and place, and he had decreased gross motor strength. The regional Poison Control Center was consulted and recommended monitoring. Intravenous ondansetron and two liters of normal saline were administered to the patient. The initial electrocardiogram (ECG) ordered to check for findings suggestive of ST-segment elevation myocardial infarction (STEMI), non-ST-elevation myocardial infarction (NSTEMI), arrhythmias, and tricyclic antidepressant toxicity, revealed the following: QRS of 120 m/s (which is new from 98 m/s in 2017); terminal R-wave >3 mm in aVR (Figure 1). The urine drug screen, which was ordered to screen for illicit drugs and coingestants, was only positive for benzodiazepines, and the comprehensive metabolic panel, which was ordered to check for electrolyte derangements, was only remarkable for a sodium concentration of 125 mmol/L, most likely due to the patient taking sacubitril/valsartan, an angiotensin-converting enzyme inhibitor, and angiotensin II receptor antagonist. The high-sensitivity troponin test, which was ordered in conjunction with the ECG to rule out a STEMI and NSTEMI, was within normal limits. 

Confirmation of the patient actually taking tianeptine via send-out labs for specialized testing was not performed. The patient was admitted to medicine for telemetry monitoring and management of hyponatremia and acute intoxication given the anticholinergic toxidrome manifestations versus opioid-like withdrawal, as the last use was approximately 12 hours prior to arrival and the Clinical Opiate Withdrawal Scale (COWS) was moderate.

Twice daily ECGs, bladder scans every four hours for urinary retention, and polyethylene glycol 3350 administration for constipation were part of the patient’s inpatient management. His home dose of 0.5 mg per os alprazolam was started for supportive care of his agitation, and four amps of sodium bicarbonate total were administered during his two-day hospital stay. The initial 2 amps of bicarbonate lowered the QRS interval to 112 m/s, but the QRS interval would fluctuate between 112 and 122 m/s. Many repeat ECGs of the patient revealing QRS prolongation were most likely due to chronic hyponatremia due to sacubitril/valsartan. On the second day of admission, the patient reported improvement in symptoms and refused further workup for hyponatremia, stating it was chronic. He was discharged with the recommendation of outpatient follow-up with a nephrologist for chronic hyponatremia and a primary care doctor regarding his drug use disorder.

## Discussion

This case highlights the complex nature of differentiating between tianeptine withdrawal and intoxication in the ED, underscoring the need for healthcare providers to be vigilant in recognizing and treating such cases. This discussion will delve deeper into the existing literature, reviewing the clinical features, management strategies, and broader public health implications of tianeptine misuse.

A case has been reported of tianeptine misuse leading to toxic leukoencephalopathy, which ultimately led to death [1, 4]. During a research investigation spanning from 2015 through 2020, which involved an analysis of calls made to Poison Control, it was found that out of a total of 48 cases, 17 individuals were determined to be experiencing acute tianeptine intoxication, while 31 cases were clearly experiencing tianeptine withdrawal. In one instance of withdrawal, a patient had used tianeptine for two weeks, taking 10 to 15 pills daily, with a notable reliance on the substance every four to six hours to prevent withdrawal symptoms [5]. In a separate study conducted between 2000 and 2017, tianeptine withdrawal reported to Poison Control included symptoms such as agitation (33.3%), nausea (33.3%), vomiting (19%), tachycardia (19.1%), hypertension (14.3%), diarrhea (9.5%), tremors (9.5%), and excessive sweating (9.5%). On the other hand, the symptoms associated with acute tianeptine toxicity closely mirrored those of withdrawal, including tachycardia, hypertension, agitation, nausea, vomiting, and diarrhea. However, acute toxicity also presented with conduction delays, drowsiness, confusion, and coma, which were not observed in withdrawal cases. Specific symptoms that were more unique to withdrawal included tremors and excessive sweating [1].

The management of acute intoxication can involve the use of sedative approaches like benzodiazepines and dexmedetomidine. In contrast, individuals going through withdrawal symptoms received treatment that included opioids alongside benzodiazepines and dexmedetomidine [5]. Additionally, positive outcomes have been achieved through the use of immediate countermeasures such as naloxone coupled with intubation, when necessary, as well as the controlled reduction of tianeptine dosage through benzodiazepines or scheduled tapers for detoxification. The most commonly applied treatments for tianeptine withdrawal encompassed benzodiazepines (57.1%), intravenous fluids (38.1%), and antiemetics (19.1%) [1]. A study demonstrated the effectiveness of a combination of buprenorphine and naloxone in assisting individuals with tianeptine use disorder to discontinue their use and maintain abstinence from tianeptine [6]. However, the potential for exacerbation of anticholinergic symptoms, such as constipation and urinary retention, must be carefully considered when using these agents.

The ECG findings in this case, indicative of tricyclic antidepressant toxicity, align with previous reports and provide a useful diagnostic clue for clinicians [7]. Recognizing these ECG patterns can prompt timely intervention and appropriate management in the ED since one cannot confirm tianeptine withdrawal without objective proof that the patient has tianeptine in the bloodstream, which was not obtained in this case. Interventions and appropriate management include using sodium bicarbonate for QRS prolongation, as demonstrated in this patient's care. The literature also underscores the need for increased regulatory oversight and public awareness regarding tianeptine. Given the rising number of cases of misuse and the significant health risks associated with its use, some states, including Michigan, Alabama, Ohio, Georgia, and Tennessee, have implemented bans on the sale of tianeptine [8]. 

Despite its therapeutic benefits, long-term use of tianeptine has generally been reported to have minimal adverse effects on cognitive functions, psychomotor activity, cardiovascular function, sleep, and weight [9]. However, reports of high-dose use and dependence, particularly among individuals with a history of substance abuse, highlight the risk of tolerance and the potential for misuse [10]. These cases often involve increasing doses to maintain the desired effects, leading to a range of physical and psychological withdrawal symptoms upon cessation.

## Conclusions

Tianeptine's complex mechanism of action, the similarity of its withdrawal symptoms to other substance use disorders, and the inability to confirm the presence of tianeptine in the blood through immediately available blood testing in the ED pose diagnostic and therapeutic challenges. Regulatory measures and clinician education are essential to addressing the public health concerns posed by tianeptine. Future research should focus on developing standardized treatment protocols and exploring the long-term outcomes of individuals affected by tianeptine use disorder.
